# Primary Hydatid Cysts in the Extremities: A Systematic Review of the Literature

**DOI:** 10.7759/cureus.63174

**Published:** 2024-06-26

**Authors:** Anna Paspala, Evgenia Mela, Michail Vailas, Konstantinos Nastos, Dionysios Dellaportas, Stylianos Kykalos, Nikolaos Machairas, Dimitrios Schizas

**Affiliations:** 1 Department of Surgery, Evgenideio Hospital, Athens, GRC; 2 First Department of Surgery, Laiko General Hospital, Athens, GRC; 3 Third Department of Surgery, Attikon University Hospital, Athens, GRC; 4 Second Propaedeutic Department of Surgery, Laiko General Hospital, Athens, GRC

**Keywords:** rare, management, extremities, echinococceal cyst, hydatid cyst

## Abstract

Primary hydatid cysts (PHCs) in the extremities are uncommon, presenting in the majority of cases with atypical clinical features. Radical surgical excision remains the mainstay of treatment. The aim of our study was to accumulate the already published data on PHCs in the extremities in terms of demographic, diagnostic, and therapeutic aspects. Three electronic databases were meticulously searched for articles published until 2024. A total of 85 studies comprising 118 patients were finally included in our review. Sixteen patients (13.5%) were diagnosed with a hydatid cyst in their upper extremity, 94 (79.7%) with a PHC in the lower extremity, and eight (6.8%) with an echinococcal cyst in the axilla. Pain and swelling were the most frequent symptoms, whereas only two patients were completely asymptomatic. The mean lesion size was 11.6 ± 7.1 cm. Preoperative serology investigation was reported in 82 out of 118 (69.5%) patients; among them, 33 (44.6%) cases had a positive preoperative serology test. The vast majority of patients (96.6%) were treated with an interventional procedure either surgical or radiological, and only seven experienced postoperative complications. No anaphylactic reaction was described perioperatively. Although preoperative diagnosis of PHCs is challenging, they should be considered in the differential diagnosis of soft tissue lesions. Treatment strategies should be individualized on a patient basis, while radical surgical excision remains the gold standard treatment.

## Introduction and background

A hydatid cyst is a zoonotic larval infection caused by Echinococcus granulosus, endemic in regions of livestock husbandry, Mediterranean countries, the Middle East, India, and Oceania [1]. Hydatid disease is considered a critical public health problem, especially in developing countries, where patients’ education and control programs are lacking. Moreover, the increasing mobility of the population due to emigration and trade favors the appearance of hydatid disease in non-endemic countries, where it is not prevalent [2].

The majority of primary hydatid cysts (PHCs) are located in the liver (65%) or the lungs (approximately 30%) [3]. Less common locations include the kidneys, spleen, bones, and brain [3]. Regarding other extremely rare sites of PHCs such as extremities, muscles, and soft tissue, only case reports and small case series have been described so far in the medical literature, even in endemic countries. Involvement of muscles is reported with a variable incidence ranging from 1 to 5%; intramuscular hydatid cyst cases usually occur due to the spread of the cyst from another area, or secondary to surgical resection of cysts from a different location. The precise mechanisms of transmission and development of PHCs in these rare locations remain unclear, although various hypotheses have been proposed.

The most common clinical manifestation is a painless growing mass, even if superimposed inflammation features have occasionally been described [4-6]. Regarding the diagnosis of PHCs in the extremities, a plethora of imaging modalities are used, but ultrasonography (U/S) and magnetic resonance imaging (MRI) seem to be the most accurate tools to achieve a reliable preoperative diagnosis [4-6]. Atypical clinical and radiological manifestations make the diagnosis of PHCs challenging, whilst in several cases diagnosis of PHCs might only be confirmed by preoperative biopsy or after surgery. Surgical resection of the entire lesion is the treatment of choice in the vast majority of cases, while attention should be focused on risks related to spillage during surgery, such as perioperative anaphylactic reactions, and recurrence.

This systematic review aims to compile and evaluate the current evidence on PHCs in the extremities, with emphasis on the demographic, diagnostic, and therapeutic aspects of this rare condition.

## Review

Materials and methods 

Study Design 

This systematic review included all relevant observational studies and case reports involving patients diagnosed with primary hydatid cysts in the extremities, whether diagnosed pre- or postoperatively. Animal studies and reviews were excluded, and only English-language studies were considered. The review encompassed patients who received either conservative or interventional treatment for primary hydatid cysts in the extremities, as well as those who did not receive any treatment. However, patients with recurrent hydatid cyst lesions in the extremities were excluded. AP and EM conducted independent and thorough literature searches, removed duplicates, and organized the selected studies into structured formats.

Search Strategy and Data Collection 

**Figure 1:**
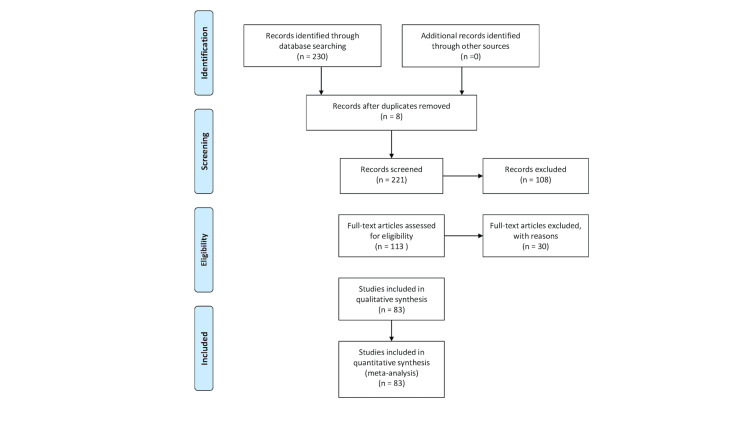
The Preferred Reporting Items for Systematic Reviews and Meta-Analyses (PRISMA) flow diagram

We conducted a systematic literature search for articles published up to April 2024 using PubMed (1966-2024), Scopus (2004-2024), and Google Scholar (2004-2024) databases, along with references from fully retrieved articles. The search employed the following keywords: “extremities,” “limbs,” “hydatid cyst,” and “echinococcal cyst.” A minimal number of keywords were used to ensure a manageable number of search results while minimizing the risk of missing relevant articles. Articles meeting or likely to meet the inclusion criteria were retrieved, specifically those describing cases of patients over 18 years old who were treated for hydatid cysts in the extremities diagnosed pre- or post-operatively. The stages of article selection are illustrated in the Preferred Reporting Items for Systematic Reviews and Meta-Analyses (PRISMA) flow diagram (Figure 1).

Data on patient characteristics included age, sex, lesion site, primary signs and symptoms, serology examination, size and type of hydatid cyst, and outcomes of clinical examination and imaging of the recruited patients. The outcomes of the study, type of treatment, pre- and post-treatment preparation, recurrence, and time of follow-up were appraised, if available. 

Definitions 

Extremities as anatomical regions include every part of the upper and lower extremities, axilla, and gluteal region.

Results

Main Characteristics of the Included Studies 

**Figure 2:**
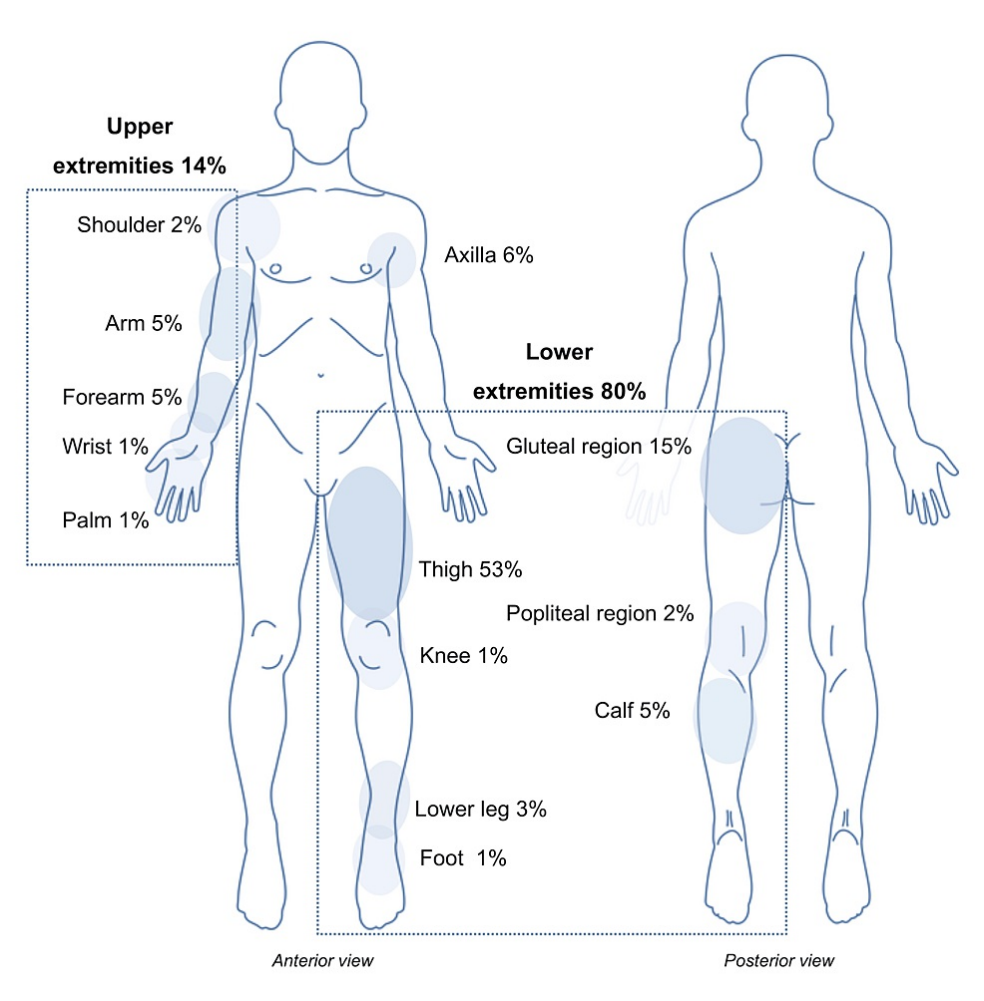
The sites of PHCs in all included patients PHCs: Primary hydatid cysts Image credit: Authors

A total of 85 studies (74 case reports and 11 case series), which comprised 118 patients, who were diagnosed with PHCs in the extremities were included in the present systematic review [1,4-85]. Table 1 shows the main characteristics of the included patients. The median age of the included patients was 42,5 years, while some 61 (51.7 %) patients were female. The male-female ratio was 1:1. Sixteen (13.5%) were diagnosed with a hydatid cyst of the upper limb, 94 (79.7%) with an HC of the lower extremity, and 8 (6.8%) with an echinococcal cyst of the axilla. Figure 2 schematically shows the sites of PHCs in all 118 included patients.

Disease-Related Characteristics

Primary symptoms and signs were reported in 109 (92.4%) cases. Among them, pain was the primary symptom in 50 patients (45.9%), swelling in 43 patients (39.4%), pruritus in four patients (3.7%), whereas only four patients (3.7 %) were asymptomatic and were incidentally diagnosed. Additionally, clinical examination revealed a palpable mass in 61 (56%) cases, neurological complications in seven cases (6.4%) and compartment syndrome in one case (0.9%).

Table 1 presents the imaging studies that were used according to preoperative evaluation, including classic X-rays (XR), U/S, computed tomography (CT), MRI, U/S-guided fine-needle aspiration (FNA), biopsy, or a combination of them. More precisely, XR, US, CT, and MRI were used in 35 (29.7%), 53 (44.9%), 36 (30.5%) and 91 (77.1%) patients, respectively. Preoperatively, twelve (10.2%) patients were diagnosed with either biopsy or U/S-guided FNA. Preoperative serology investigation was reported in 74 out of 118 (62.7%) patients. Among them, 33 (44.6%) cases had a positive preoperative serology test. Imaging modalities described the specific type of hydatid cyst in 90 (76.3%) patients. More specifically, 74 (82.2%) out of 90 were diagnosed with a multilocular lesion, while 16 (17.8%) had a unilocular lesion. Preoperative diagnosis of the lesion through biopsy, radiological studies, or patients’ history was available for 110 (93.2%) cases. Among them, 98 (89.1 %) patients were diagnosed with hydatid cysts, whereas preoperative diagnosis was abscess or tumor in nine patients (8.2%), hematoma in two patients (1.8 %) and sarcoma in one (0.9%) patient. Concerning the size of lesions, data was available in 70.3% of cases; the mean size of lesions in all the included patients was 11.6±7.1cm. As depicted in Table 2, hydatid cyst-specific preoperative preparation was reported in 37 (44.6%) cases. More specifically, albendazole was administrated in 35 (94.6 %) patients, mebendazole in one (2.7%) patient and no-specific anthelmintic drugs in one (2.7%) case.

**Table 1:** Main characteristics of the included patients NA: Not available; std: standard deviation

Patient Characteristics	Number of Patients
Total number of patients	n=118
Sex	61 Female
Age (median, years)	42.5
Primary symptoms (n=109)	
No symptoms	2
Swelling	43
Pain	50
Palpable mass	61
Pruritus	4
Neurological complications	7
Compartment syndrome	1
Diagnostic methods (n=118)	
None	5
XR	35
US	53
CT	36
MRI	91
US-guided aspiration	7
Biopsy	5
Serology examination (n=81)	
Yes	74
No	7
Positive	33
Negative	23
NA result	18
Size (cm, mean ±std)	11.6 ± 7.1
Cyst type (n=90)	
Multilocular	74
Unilocular	16
Preoperative diiagnosis (n=110)	
Hydatid cyst	98
Hematoma	2
Abscess or tumor	9
Sarcoma	1

**Table 2:** Perioperative data and outcomes PAIR: Puncture, aspiration, injection, and reaspiration

Treatment (n=117)	
PAIR	5
PAIR & Surgical excision	2
Radical Surgical excision	35
Surgical excision	72
No surgical treatment	3
Postoperative complications (n=54)	
None	47
Abscess or Infection	3
Seroma	1
Hematoma	1
Cellulitis	1
Hepatic toxicity	1
Preoperative treatment (n=83)	
None	46
Albendazole	35
Mebendazole	1
Anthelmintic drugs	1
Postoperative treatment (n=84)	
Albendazole	66
Mebendazole	3
None	14
Anthelmintic drugs	1
Recurrence (n=93)	
Yes	2
No	91
Follow-up (months, median)	20

Treatment Outcomes 

Data regarding treatment outcomes were available for 117 (99.2%) patients. More specifically, nearly all patients (97.4%) underwent an interventional procedure either surgical or radiological for the treatment of their hydatid cysts except from three cases. Among these in three (2.6%) cases, one died before the implementation of any treatment and two received conservative treatment with albendazole. Regarding cases who underwent an interventional approach, 72 (63.1%) had a surgical excision, 35 (30.7%) a radical surgical excision, whereas a combination of the puncture, aspiration, injection, and reaspiration (PAIR) technique with surgical excision and only PAIR treatment was described in two (1.8 %) and five (4.4%) cases, respectively. 

Postoperative outcomes were reported in 54 (45.8%) patients. No postoperative complications were described in the majority (87%) of cases, while seven (13%) patients developed complications. Among them, abscess or infection presented in three (42.9%) cases, one patient developed a seroma, one developed a hematoma, one developed hepatic toxicity and one case was complicated with cellulitis. As depicted in Table 2, hydatid cyst-specific postoperative therapy was reported in 70 (59.3%) cases. More specifically, albendazole was administrated in 66 (94.3%) patients, mebendazole in three (4.3%) patients, and a not otherwise specified anthelmintic drug in one case (1.4%). Ninety-three (78.8%) cases reported outcomes concerning a possible recurrence of hydatid cysts after the first treatment. However, only two (2.2%) patients relapsed. No postoperative deaths were reported from the included studies. The median follow-up was 20 (2-170) months.

Discussion

Echinococcosis is a zoonosis caused in humans by the larval stage of cestodes, belonging to the genus Echinococcus granulosus of the family Taeniidae [86]. While other forms of echinococcosis are rare but severe, and sometimes life-threatening, cystic echinococcosis is endemic and treatable [1,87]. PHCs in the extremities are a rare condition and are occasionally challenging for clinicians in terms of diagnosis and management. Our study shows that both age and sex distribution of PHCs in the extremities are similar to those of hepatic hydatid cysts [86]. 

The precise mechanism of development of PHCs in the extremities remains ill-determined. Two potential mechanisms have been suggested: (a) direct contamination through injured skin or (b) subcutaneous colonization of ingested eggs after passing through the liver and lungs. Our systematic review showed that PHCs in the lower extremities were more common compared to PHCs in upper extremities and especially hands. Similar outcomes were reported by Kayaalp et al., who also mentioned that the direct contact theory was considered unlikely to be the dominant mechanism for the development of PHCs [86]. As a result, subcutaneous colonization of the parasite in the circulation after ingestion might be a more reliable mechanism than the direct contact theory [86]. Furthermore, since the gastrointestinal environment is required to transform eggs into larvae, theories involving mechanisms other than egg ingestion are not plausible [86]. Abhishek et al.proposed the theory that parasites might flee the portal-liver tough filter by using lymphatic or venous shunts directly to systemic circulation [88].

The majority of patients presented with atypical clinical manifestations including a palpable, mostly mobile lesion, pain, and swelling. Several imaging studies have been used to establish the exact diagnosis for these lesions. CT, MRI, and U/S in experienced hands are the most useful modalities to diagnose PHCs in the extremities as they can efficiently recognize specific features, including thick cyst wall, calcifications, daughter cysts, and a germinative membrane separate from the cyst wall. Although routine laboratory tests do not show unique changes in PHCs in the extremities, serology examination for a suspicious PHC might be necessary for the differential diagnosis, which includes abscess, sebaceous cyst, lipoma, tuberculous abscess, hernia, sarcoma, chronic hematoma, synovial cyst, and necrotic soft tumor. Considering the possibility of intraoperative anaphylactic reactions and high recurrence rate, preoperative diagnosis might play a crucial role, but as our study reported no anaphylactic reaction occurred and only two patients presented with recurrence after surgical excision. Interestingly, Kayaalp et al. described that, despite the fact that only 45% of the included patients had an accurate preoperative diagnosis, none presented with either early or late complications such as anaphylaxis or recurrence. Previous studies have demonstrated a potential role in preoperative diagnosis using FNA in extra-abdominal cases of PHCs [89]. We highlighted that only 12 cases underwent preoperative FNA, and 33 patients had positive preoperative serologic tests. Similarly, in the study conducted by Kayaalp et al., only five patients had preoperative FNA, while serologic tests were usually negative [86]. Based on these data, although preoperative FNA has an unclear role, it is also a safe procedure without significant risk of anaphylactic reaction and spillage [86,89].

According to our review, surgical excision of the lesion was the most widely implemented treatment for PHCs of the extremities. Several disadvantages follow the traditional surgical approach, such as the necessity for extensive surgical resection in some cases and the need for general anesthesia. Preoperative and adjuvant administration of anthelminthic medication are widely used in treatment protocols. Percutaneous treatment is widely employed for most of liver hydatid cysts as a safe and effective alternative treatment with favorable results [90]. The percutaneous approach has also been reported as an effective treatment strategy for PHCs in the lungs, kidneys, orbita, and parotid glands; however, there are no studies in the literature describing the results of percutaneous treatment of PHCs in the extremities [91]. According to our results, PAIR was applied to only five cases, while two patients underwent a combination of PAIR and surgical excision [90]. Since only a few cases exist in the literature for percutaneous treatment of PHCs in the extremities, the embracement of this procedure in clinical practice as the treatment of choice is not yet recommended.

To the best of our knowledge, this study is the only one in the literature that provides a comprehensive report on the natural history, characteristics, and management of adults with PHCs in the extremities. An extensive literature search, with no date restrictions, minimized the risk of missing relevant articles. The exact prevalence of PHCs in the extremities remains unclear, and information on their pathophysiology, clinical presentation, and treatment is limited to case reports and small case series. This rarity prevents further analysis. Additionally, the significant heterogeneity among the included studies and the omission of certain parameters by some studies pose further limitations to our review.

## Conclusions

In conclusion, preoperative diagnosis of PHCs in the extremities can be challenging due to varying radiologic features and clinical symptomatology. Nonetheless, it should be considered in the differential diagnosis of a painless mass located in extremities especially in patients with a specific history and radiological characteristics. Management options should be individualized on a patient-to-patient basis. Infectious disease specialists, surgeons, anesthetists, and potentially interventional radiologists could all weigh in on deciding what could be the best management option for the patient. Radical surgical excision remains the treatment of choice for PHCs in the extremities and is accompanied by favorable outcomes concerning morbidity, mortality, symptom relief, and lesion recurrence.
